# Comparison of publicly available artificial intelligence models for pancreatic segmentation on T1-weighted Dixon images

**DOI:** 10.1007/s11604-025-01814-5

**Published:** 2025-06-18

**Authors:** Yuki Sonoda, Shota Fujisawa, Mariko Kurokawa, Wataru Gonoi, Shouhei Hanaoka, Takeharu Yoshikawa, Osamu Abe

**Affiliations:** 1 Department of Radiology, Graduate School of Medicine, The University of Tokyo, 7-3-1 Hongo, Bunkyo-ku, Tokyo, 113-8655 Japan; 2 Department of Computational Diagnostic Radiology and Preventive Medicine, The University of Tokyo Hospital, 7-3-1 Hongo, Bunkyo-ku, Tokyo, 113-8655 Japan

**Keywords:** Pancreas, Dixon method, Intrapancreatic fat fraction, Artificial intelligence, Pancreatic fat deposition, Segmentation

## Abstract

**Purpose:**

This study aimed to compare three publicly available deep learning models (TotalSegmentator, TotalVibeSegmentator, and PanSegNet) for automated pancreatic segmentation on magnetic resonance images and to evaluate their performance against human annotations in terms of segmentation accuracy, volumetric measurement, and intrapancreatic fat fraction (IPFF) assessment.

**Materials and methods:**

Twenty upper abdominal T1-weighted magnetic resonance series acquired using the two-point Dixon method were randomly selected. Three radiologists manually segmented the pancreas, and a ground-truth mask was constructed through a majority vote per voxel. Pancreatic segmentation was also performed using the three artificial intelligence models. Performance was evaluated using the Dice similarity coefficient (DSC), 95th-percentile Hausdorff distance, average symmetric surface distance, positive predictive value, sensitivity, Bland–Altman plots, and concordance correlation coefficient (CCC) for pancreatic volume and IPFF.

**Results:**

PanSegNet achieved the highest DSC (mean ± standard deviation, 0.883 ± 0.095) and showed no statistically significant difference from the human interobserver DSC (0.896 ± 0.068; *p* = 0.24). In contrast, TotalVibeSegmentator (0.731 ± 0.105) and TotalSegmentator (0.707 ± 0.142) had significantly lower DSC values compared with the human interobserver average (*p* < 0.001). For pancreatic volume and IPFF, PanSegNet demonstrated the best agreement with the ground truth (CCC values of 0.958 and 0.993, respectively), followed by TotalSegmentator (0.834 and 0.980) and TotalVibeSegmentator (0.720 and 0.672).

**Conclusion:**

PanSegNet demonstrated the highest segmentation accuracy and the best agreement with human measurements for both pancreatic volume and IPFF on T1-weighted Dixon images. This model appears to be the most suitable for large-scale studies requiring automated pancreatic segmentation and intrapancreatic fat evaluation.

**Supplementary Information:**

The online version contains supplementary material available at 10.1007/s11604-025-01814-5.

## Introduction

Research on pancreatic imaging is diverse; in particular, pancreatic segmentation has promising applications in various contexts. Accurate segmentation facilitates the detection of morphological changes and the analysis of quantitative features recently associated with or causally linked to pancreatic diseases, including pancreatic cancer [1–5]. For example, a retrospective study of pre-diagnostic computed tomography (CT) images from 41 patients with pancreatic cancer found a localized “K-shaped” contraction in the pancreatic parenchyma in 58.5% of cases, compared with 0.16% in the control group [6].

Intrapancreatic fat deposition has been hypothesized to contribute to the development of pancreatic diseases, including pancreatitis and pancreatic cancer, through mechanisms such as inflammation and lipotoxicity [7]. Large-scale cohort studies have further shown that intrapancreatic fat deposition is a risk factor for pancreatic cancer and is also associated with acute pancreatitis and diabetes mellitus [1].

Magnetic resonance imaging (MRI) using the Dixon method can assess both morphology and fat content, making Dixon-based approaches valuable for pancreatic imaging [1–4]. However, accurate measurements often require time-consuming manual segmentation of the entire pancreas or the placement of arbitrarily defined regions of interest, posing significant challenges, particularly for large-scale datasets. Fortunately, artificial intelligence (AI) techniques enable automated segmentation, addressing these limitations.

Several AI models are publicly available for segmenting abdominal organs in general MRI studies. Among these, TotalSegmentator MRI automatically segments 59 anatomical structures in MRI without relying on a specific sequence. It was developed using 298 MRI and 227 CT images within the nnU-Net framework [8]. TotalVibeSegmentator was designed to segment 71 anatomical structures in whole-body MRI using data from the German National Cohort and UK Biobank, also based on the nnU-Net architecture [9]. PanSegNet, a hybrid model combining nnU-Net with a transformer self-attention mechanism, was trained on 1,350 publicly available CT scans and 767 MRI scans from five medical centers [10].

Although TotalSegmentator, TotalVibeSegmentator, and PanSegNet have demonstrated utility in pancreatic segmentation on test datasets, their performance on Dixon MRI for both morphological and quantitative assessments remains unclear. Moreover, standard image acquisition with the Dixon method generates multiple image types (in-phase, opposed-phase, water-only, and fat-only), and the optimal input for each model is uncertain. Furthermore, limited evidence is available on how these deep learning-based automatic segmentation models compare with manual segmentation by humans.

This study had two objectives: to compare the performance of three publicly available deep learning models (TotalSegmentator, TotalVibeSegmentator, and PanSegNet) for pancreatic segmentation using different types of T1-weighted Dixon images, and to evaluate the concordance, bias, and variability of pancreatic volume and intrapancreatic fat fraction (IPFF) measurements against the manually derived ground truth.

## Materials and methods

Twenty abdominal MRI examinations performed between 2013 and 2023 were selected from our institution’s database. This study was conducted as a secondary use of retrospective data originally derived from a patient cohort that underwent liver function assessments, extracted from a separate study focusing on liver conditions. The database contained 1090 cases which included T1-weighted two-point Dixon images (in-phase, opposed-phase, water-only, and fat-only). The study protocol was approved by the ethics committee of the University of Tokyo Hospital (Approval No. 2561). This retrospective study was conducted in accordance with the principles of the Declaration of Helsinki. The need for informed consent was waived because of the retrospective nature of the study.

To achieve diversity in the dataset, we ensured the following conditions were met. First, for imaging equipment, we selected five cases from each combination of 1.5 T and 3.0 T GE HealthCare (GE; Signa HDx) and 1.5 T Siemens (Avanto) and 3.0 T Siemens (Skyra) scanners. Second, for imaging timeframe, ten cases were chosen from 2013–2017 and ten from 2018–2023 to reflect variations in image quality owing to improvements in scanner technology over time. Third, for age distribution, at least one patient from each decade of life (20 s, 30 s, 40 s, 50 s, 60 s, 70 s, and 80 s) was included to ensure age variability, as age-related pancreatic changes, particularly fat infiltration, are well-documented [1].

The sampling procedure was conducted by randomly selecting five candidates from each scanner type and repeating this selection process until the additional predefined criteria for imaging timeframe and age distribution were satisfied. The database from which we sampled contained 1090 cases distributed as follows: 122 cases on 1.5 T GE, 712 cases on 3.0 T GE, 20 cases on 1.5 T Siemens, and 236 cases on 3.0 T Siemens scanners. With respect to imaging timeframes, 576 cases were from 2013–2017 and 514 cases from 2018–2023. The age distribution in the database was: 39 cases aged 20–29 years, 47 cases aged 30–39 years, 107 cases aged 40–49 years, 189 cases aged 50–59 years, 284 cases aged 60–69 years, 322 cases aged 70–79 years, and 96 cases aged 80–89 years.

Among the selected 20 subjects, the mean age was 58.7 ± 14.2 years, with 14 males and 6 females. Three patients had type 2 diabetes, but none had other known pancreatic diseases at the time of examination. The average body mass index was 23.8 ± 4.5 kg/m^2^. Each MRI scan covered the entire pancreas.

Dixon imaging separates water and fat signals by exploiting phase shifts caused by chemical shift differences [11], with water-only and fat-only images generated by processing the acquired in-phase and opposed-phase signals. Opposed-phase imaging parameters were as follows: repetition time ranged from 3.94 to 11.90 ms, echo time from 1.18 to 2.39 ms, and slice thickness from 1.50 to 5.00 mm. Image matrix dimensions ranged from 192 to 512 rows and from 256 to 512 columns, with an in-plane resolution of 0.625–1.562 mm in both *X* and *Y* directions. The in-phase echo time was twice the opposed-phase echo time, whereas all other imaging parameters were identical to those of the opposed-phase sequence. All scans were obtained without contrast or before contrast administration.

Three radiologists manually segmented the pancreas using 3D Slicer (version 5.6.2; Segment Editor module). Segmentation was performed using the standard brush tool, and interpolation was not used. The group included a board-certified radiologist with 8 years of experience (observer 1) and two radiology residents with 3 and 2 years of experience (observers 2 and 3, respectively). Each radiologist independently segmented the pancreas on the water-only images, which provide good contrast between the pancreatic parenchyma and surrounding fat [11]. A ground-truth reference mask was generated through a majority vote on a voxel-wise basis, where any voxel labeled as the pancreas by at least two observers was considered part of the pancreas.

We evaluated three publicly available deep learning models—TotalSegmentator MRI (version 2.5.0), TotalVibeSegmentator (version 1.0.0), and PanSegNet (T1-weighted version). Each model was provided with four sets of Dixon images per case: in-phase, opposed-phase, water-only, and fat-only.

Volumes were calculated from each segmentation mask. To measure the IPFF, we first applied an erosion operation to the pancreas mask to minimize boundary effects [1], then computed the mean fractional fat value for each voxel within the mask using the following formula:

1 $${\text{fractional-fat}} = \frac{{\text{signal intensity }(\text{SI})\text{ on fat-only image}}}{{({\text{SI on water-only image}} + {\text{SI on fat-only image}})}}.$$

Segmentation performance was evaluated by comparing AI-generated masks with the ground truth using the Dice similarity coefficient (DSC) [12], 95th-percentile Hausdorff distance (HD95), average symmetric surface distance (ASSD), positive predictive value (PPV), and sensitivity. We also calculated the average DSC, HD95, and ASSD values for the three human observers. Paired *t* tests (*α* = 0.05) were used to compare the DSC, HD95, and ASSD between AI-generated masks and the ground truth with those from human interobserver segmentation. For comparisons between human interobserver and AI-ground-truth segmentations, the input image type (in-phase, opposed-phase, fat-only, or water-only) that produced the highest score for each AI model was selected.

Bland–Altman plots were used to visualize bias and 95% limits of agreement for volumetric and IPFF measurements. To assess agreement between AI-derived measurements and the ground truth for both volume and IPFF, we computed the concordance correlation coefficient (CCC), a statistical measure of agreement ranging from − 1 to 1. Values closer to 1 indicate higher concordance between two measurement methods. The CCC is defined as:

$$\rho_{c} = \frac{{2\rho \sigma_{x} \sigma_{y} }}{{\sigma_{x}^{2} + \sigma_{y}^{2} + (\mu_{x} - \mu_{y} )^{2} }}$$

where *µ*_*x*_ and *µ*_*y*_ are the means of the two measurement methods, *σ*_*x*_ and *σ*_*y*_ are their standard deviations, and *ρ* is the Pearson correlation coefficient between them [13]. A CCC of 0.95 or higher is generally considered strong agreement between measurements [14]. The CCC was calculated for each AI model and the ground truth for both pancreatic volume and IPFF. All evaluations were performed in the original voxel space. Continuous variables are reported as means ± standard deviations.

## Results

**Fig. 1:**
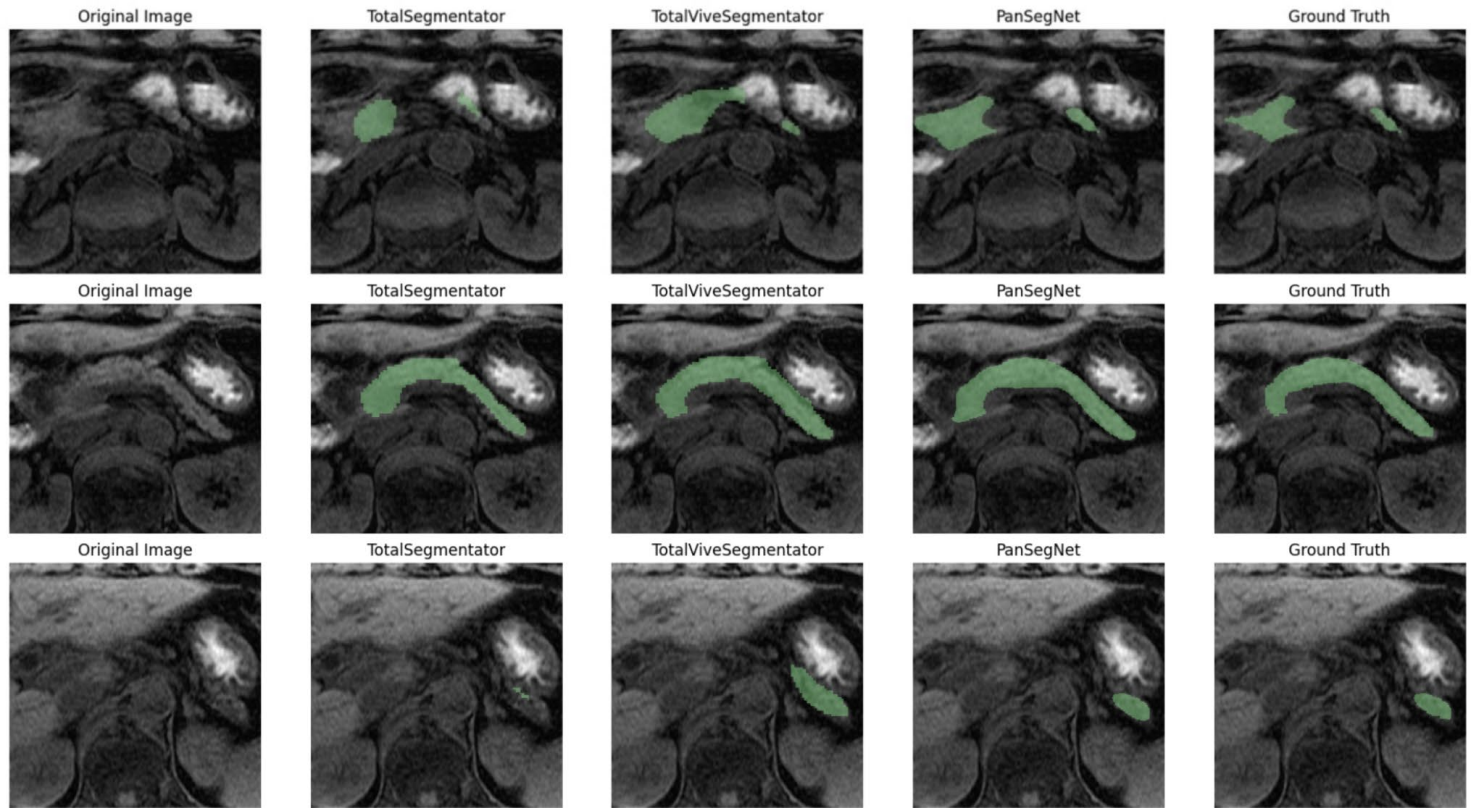
Example of segmentations from artificial intelligence models (TotalSegmentator, TotalVibeSegmentator, and PanSegNet) compared with the ground truth. Water-only images were acquired using the two-point Dixon method on a 1.5 T scanner with a slice thickness of 3.0 mm, repetition time of 6.304 ms, and echo time of 2.084 ms. The slices include the pancreatic head, body, and tail

Examples of segmentation results from the AI models and the ground truth are shown in Fig. 1.

Table 1 summarizes the DSC, HD95, ASSD, PPV, and sensitivity for each model compared with the ground truth across four image types (in-phase, opposed-phase, water-only, and fat-only). Both PanSegNet and TotalSegmentator achieved their highest DSC on water-only images, whereas TotalVibeSegmentator showed fairly consistent but lower performance across all four Dixon-derived image types. PanSegNet achieved the highest DSC (0.883 ± 0.095), followed by TotalVibeSegmentator (0.731 ± 0.105) and TotalSegmentator (0.707 ± 0.142) on water-only images.

**Table 1:** Performance metrics for each model across different image types

Model	Metric	In-phase	Opposed-phase	Water-only	Fat-only
TotalSegmentator	DSC	0.466 ± 0.215	0.616 ± 0.180	**0.707 ± 0.142**	0.454 ± 0.277
TotalVibeSegmentator		0.720 ± 0.116	0.721 ± 0.105	**0.731 ± 0.105**	0.718 ± 0.109
PanSegNet		0.100 ± 0.197	0.363 ± 0.328	**0.883 ± 0.095**	0.000 ± 0.000
TotalSegmentator	HD95 [mm]	29.111 ± 27.755	17.032 ± 9.996	**10.743 ± 6.397**	45.068 ± 41.768
TotalVibeSegmentator		10.276 ± 4.650	10.003 ± 5.264	**9.596 ± 4.997**	10.135 ± 5.269
PanSegNet		(65.774 ± 32.214)	(43.216 ± 34.817)	**6.272 ± 6.890**	(75.033 ± 22.395)
TotalSegmentator	ASSD [mm]	6.369 ± 7.483	3.135 ± 1.934	**2.000 ± 1.161**	10.251 ± 12.154
TotalVibeSegmentator		2.244 ± 0.962	2.123 ± 0.865	**2.020 ± 0.842**	2.134 ± 0.874
PanSegNet		(21.774 ± 11.366)	(13.073 ± 11.697)	**0.916 ± 1.080**	(35.794 ± 13.676)
TotalSegmentator	PPV	0.730 ± 0.233	0.792 ± 0.116	**0.818 ± 0.089**	0.699 ± 0.355
TotalVibeSegmentator		0.638 ± 0.136	0.637 ± 0.125	**0.651 ± 0.127**	0.638 ± 0.132
PanSegNet		0.233 ± 0.330	0.420 ± 0.365	**0.853 ± 0.095**	0.000 ± 0.001
TotalSegmentator	Sensitivity	0.371 ± 0.192	0.543 ± 0.195	**0.641 ± 0.184**	0.365 ± 0.230
TotalVibeSegmentator		0.855 ± 0.113	0.855 ± 0.105	**0.859 ± 0.105**	0.846 ± 0.104
PanSegNet		0.091 ± 0.186	0.325 ± 0.305	**0.917 ± 0.109**	0.000 ± 0.000

Boldface entries indicate the best performance of each model across the four image types. Values are presented as mean ± standard deviation. Numbers in parentheses represent instances where the model did not produce segmentation masks, making the metrics undefined. In such cases, the average was calculated from the defined values only

*DSC* dice similarity coefficient, *HD95* 95th-percentile Hausdorff distance (mm), *ASSD* average symmetric surface distance (mm), *PPV* positive predictive value

Table 2 summarizes the comparison between AI segmentation and human interobserver metrics. PanSegNet showed no significant difference from the human interobserver average DSC (0.896 ± 0.068; *p* = 0.24). In contrast, TotalVibeSegmentator and TotalSegmentator had significantly lower DSC values (*p* < 0.001). Similarly, PanSegNet outperformed the other models in terms of HD95 and ASSD.

**Table 2:** Comparison of artificial intelligence segmentation with human interobserver metrics

Model	Metrics	Mean	Difference [95% CI]	*p* value
Human interobserver average	DSC	0.896 ± 0.068		–
TotalSegmentator		0.707 ± 0.142	− 0.190 [− 0.244, − 0.135]	< 0.001
TotalVibeSegmentator		0.731 ± 0.105	− 0.165 [− 0.205, − 0.125]	< 0.001
PanSegNet		0.883 ± 0.095	− 0.014 [− 0.037, 0.010]	0.242
Human interobserver average	HD95 [mm]	4.949 ± 2.286		–
TotalSegmentator		10.743 ± 6.397	5.794 [2.911, 8.676]	< 0.001
TotalVibeSegmentator		9.596 ± 4.997	4.647 [2.502, 6.791]	< 0.001
PanSegNet		6.272 ± 6.890	1.323 [− 1.461, 4.107]	0.332
Human interobserver average	ASSD [mm]	0.659 ± 0.401		–
TotalSegmentator		2.000 ± 1.161	1.342 [0.825, 1.858]	< 0.001
TotalVibeSegmentator		2.020 ± 0.842	1.361 [0.974, 1.749]	< 0.001
PanSegNet		0.916 ± 1.080	0.257 [− 0.171, 0.686]	0.224

Human interobserver average represents the mean ± standard deviation of the metrics (DSC, HD95, and ASSD) calculated among the three human observers. The *p* values were determined using paired t tests comparing each model with the human interobserver average. Differences between each artificial intelligence model and human interobserver measurements are presented with 95% confidence intervals (95% CI)

*DSC* dice similarity coefficient, *HD95* 95th-percentile Hausdorff distance (mm), *ASSD* average symmetric surface distance (mm)

**Fig. 2:**
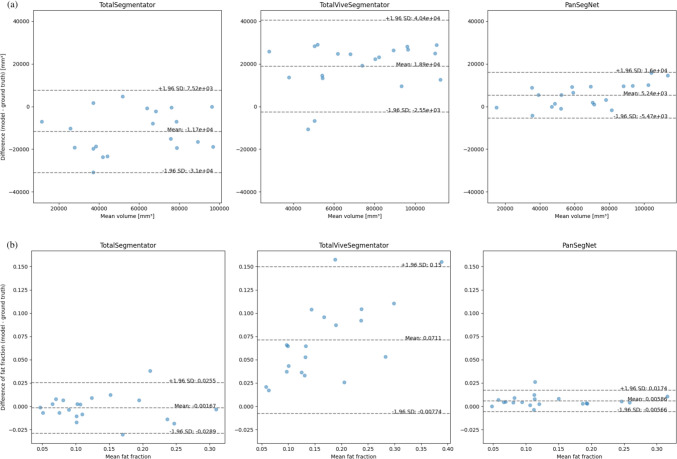
Bland–Altman plots comparing pancreatic volume measurements by artificial intelligence models with the ground truth. The plots show bias and 95% limits of agreement for each model in **a** volume estimates and **b** intrapancreatic fat fraction measurements compared with the ground truth

The ground-truth pancreatic volume was 63,000 mm^3^ across 20 subjects (Supplementary Table 1). On average, PanSegNet (+ 5240 mm^3^) and TotalVibeSegmentator (+ 18,900 mm^3^) overestimated pancreatic volumes, whereas TotalSegmentator underestimated them by − 11,700 mm^3^. Bland–Altman analysis showed that PanSegNet had narrower 95% limits of agreement compared with the other two models (Fig. 2a). PanSegNet achieved the highest CCC for pancreatic volume (0.958), followed by TotalSegmentator (0.834) and TotalVibeSegmentator (0.720; Table 3).

**Table 3:** Comparison of CCC for volumetric and intrapancreatic fat fraction measurements across the three models

Model	CCC for pancreatic volume	CCC for intrapancreatic fat fraction
TotalSegmentator	0.834	0.980
TotalVibeSegmentator	0.720	0.672
PanSegNet	0.958	0.993

*CCC* concordance correlation coefficient

The ground-truth IPFF was 0.133 ± 0.074 across 20 subjects (Supplementary Table 2). Bland–Altman analysis showed that the measurements from TotalSegmentator differed slightly from the ground truth (− 0.002), whereas PanSegNet (+ 0.006) and TotalVibeSegmentator (+ 0.071) overestimated the IPFF. PanSegNet demonstrated the narrowest 95% limits of agreement among the three models (Fig. 2b). For the CCC of IPFF measurements, PanSegNet achieved the highest value (0.993), followed by TotalSegmentator (0.980), whereas TotalVibeSegmentator was substantially lower (0.672; Table 3).

## Discussion

In this study, we evaluated three publicly available AI models for pancreatic segmentation on T1-weighted images acquired using the Dixon method. PanSegNet consistently outperformed the other two models in terms of DSC, HD95, ASSD, volume accuracy, and IPFF concordance with human segmentation.

Despite being trained primarily on contrast-enhanced T1-weighted images [11], PanSegNet performed well on non-contrast water-only images, likely because the pancreas appears with high signal intensity while surrounding fat shows low signal intensity in these images. TotalSegmentator and TotalVibeSegmentator also generally achieved their best performance on water-only images. In terms of volume measurement, both PanSegNet and TotalVibeSegmentator tended to produce larger masks, whereas TotalSegmentator produced smaller ones. For IPFF measurement, PanSegNet and TotalSegmentator were generally accurate, whereas TotalVibeSegmentator tended to overestimate fat content. This overestimation is likely explained by the larger mask including more peripancreatic fat, which inflates the average fat fraction. In contrast, a smaller mask is less likely to include peripancreatic fat, reducing its effect on the fat fraction calculation.

The average DSC among human observers did not exceed 0.9, highlighting the inherent challenge of pancreatic segmentation. The pancreas is long and narrow, with a relatively high surface area compared with its volume and unclear boundaries with adjacent tissues. Because ground-truth segmentation relies on human judgment, interobserver variability may limit the maximum achievable DSC.

For IPFF measurement, the two-point Dixon method can be affected by factors such as field inhomogeneities and T2* decay [15]. Although more advanced multiparametric approaches may offer greater accuracy, they are not always available and can be expensive. The two-point Dixon method, commonly used in clinical practice, can still provide clinically meaningful data when reliable segmentation is available. Notably, one study using a method similar to that in the present study found that the prevalence of pathological pancreatic fat infiltration measured by the two-point Dixon method closely matched that measured by magnetic resonance spectroscopy [16], suggesting that it can serve as a useful surrogate [1].

PanSegNet achieved a high CCC for both pancreatic volume and IPFF assessments, demonstrating superior concordance and consistency compared with the other models. In view of its high CCC and low variability, PanSegNet appears both reliable and consistent for these assessments. Therefore, our findings suggest that, among the three evaluated models, PanSegNet is the most suitable for large-scale analyses of the pancreas on T1-weighted Dixon images.

Our study has several limitations. Although maintaining standardized imaging conditions is essential for evaluating the reproducibility and inherent properties of AI models, testing these models in diverse environments with varying parameters and image qualities could provide valuable insights into their generalizability [17]. Achieving this balance requires a large dataset, which is difficult to obtain because manual segmentation is necessary for creating ground-truth annotations, thereby limiting the sample size—a key limitation of this study. In addition, we introduced specific sampling conditions to ensure variations in imaging conditions, scanner types, and age distributions; however, introducing multiple conditions within a limited sample size might have resulted in biases affecting segmentation performance. Furthermore, the participants in this study were individuals with suspected liver conditions rather than pancreatic disease, which may affect model performance in populations with more extensive pancreatic pathology. Although DSC, HD95, and ASSD measure global shape similarity, they do not necessarily reflect the ability to detect localized changes, such as focal atrophy near pancreatic cancer. Future studies using longitudinal data or larger, more diverse populations with a range of pancreatic pathologies may help clarify the full potential of these models. In conclusion, among the three publicly available AI models for pancreatic segmentation on Dixon MRI, PanSegNet most closely matched human annotations in segmentation accuracy, volumetric measurement, and IPFF assessment. These results suggest that PanSegNet is a reliable tool for large-scale pancreatic imaging studies requiring automated segmentation and fat quantification.

## Supplementary Information

Below is the link to the electronic supplementary material.

Supplementary file1 (DOCX 19 KB)
