# Supplementary material for: Comparison of publicly available artificial intelligence models for pancreatic segmentation on T1-weighted Dixon images
Source: Jpn J Radiol. 2025 Jun 18;43(10):1663–9. doi: 10.1007/s11604-025-01814-5 (PMC12479682; doi:10.1007/s11604-025-01814-5)
Supplement: Supplementary file 1 — Supplementary file1 (DOCX 19 KB) [file 11604_2025_1814_MOESM1_ESM.docx]

Supplementary Table 1. Results for Pancreatic Volume (mm³) Comparisons between Each Segmentation Method, Observers and Ground Truth

| Model | Volume (mm³) | Standard Deviation (mm³) | Difference　(mm³) | Standard Deviation of Differences (mm³) |
| --- | --- | --- | --- | --- |
| Ground Truth | 63,000 | 24,500 |  |  |
| PanSegNet | 68,200 | 28,100 | 5,240 | 5,610 |
| TotalViveSegmentator | 81,900 | 27,800 | 18,900 | 11,200 |
| TotalSegmentator | 51,300 | 26,400 | -11,700 | 10,100 |
| Observer 1 | 65,100 | 25,100 | 2,130 | 2,990 |
| Observer 2 | 63,100 | 24,400 | 100 | 3,690 |
| Observer 3 | 65,200 | 25,600 | 2,230 | 3,510 |

Difference = (Model Volume) - (Ground Truth Volume).

Supplementary Table 2. Results for Intrapancreatic Fat Fraction Comparisons between Each Segmentation Method and Observers and Ground Truth

| Model | Fat Fraction | Standard Deviation | Difference | Standard Deviation of Differences |
| --- | --- | --- | --- | --- |
| Ground Truth | 0.133 | 0.0735 |  |  |
| PanSegNet | 0.139 | 0.0738 | 0.0059 | 0.006 |
| TotalViveSegmentator | 0.204 | 0.101 | 0.071 | 0.041 |
| TotalSegmentator | 0.131 | 0.0731 | -0.0017 | 0.014 |
| Observer 1 | 0.135 | 0.0744 | 0.0015 | 0.003 |
| Observer 2 | 0.136 | 0.072 | 0.0028 | 0.007 |
| Observer 3 | 0.142 | 0.0775 | 0.0084 | 0.01 |

Difference = (Model Fat Fraction) - (Ground Truth Fat Fraction).
